# Microservice Security Agent Based On API Gateway in Edge Computing

**DOI:** 10.3390/s19224905

**Published:** 2019-11-10

**Authors:** Rongxu Xu, Wenquan Jin, Dohyeun Kim

**Affiliations:** 1 Department of Computer Engineering, Jeju National University, Jeju 63243, Korea; rongxu@jejunu.ac.kr; 2 Bigdata Research Center, Jeju National University, Jeju 63243, Korea; wenquan.jin@jejunu.ac.kr

**Keywords:** Internet of Things, edge computing, API Gateway, microservice, authentication

## Abstract

Internet of Things (IoT) devices are embedded with software, electronics, and sensors, and feature connectivity with constrained resources. They require the edge computing paradigm, with modular characteristics relying on microservices, to provide an extensible and lightweight computing framework at the edge of the network. Edge computing can relieve the burden of centralized cloud computing by performing certain operations, such as data storage and task computation, at the edge of the network. Despite the benefits of edge computing, it can lead to many challenges in terms of security and privacy issues. Thus, services that protect privacy and secure data are essential functions in edge computing. For example, the end user’s ownership and privacy information and control are separated, which can easily lead to data leakage, unauthorized data manipulation, and other data security concerns. Thus, the confidentiality and integrity of the data cannot be guaranteed and, so, more secure authentication and access mechanisms are required to ensure that the microservices are exposed only to authorized users. In this paper, we propose a microservice security agent to integrate the edge computing platform with the API gateway technology for presenting a secure authentication mechanism. The aim of this platform is to afford edge computing clients a practical application which provides user authentication and allows JSON Web Token (JWT)-based secure access to the services of edge computing. To integrate the edge computing platform with the API gateway, we implement a microservice security agent based on the open-source Kong in the EdgeX Foundry framework. Also to provide an easy-to-use approach with Kong, we implement REST APIs for generating new consumers, registering services, configuring access controls. Finally, the usability of the proposed approach is demonstrated by evaluating the round trip time (RTT). The results demonstrate the efficiency of the system and its suitability for real-world applications.

## 1. Introduction

As intelligent factories [1], smart cities [2], and augmented reality [3] have attracted attention from both academic and industrial researchers, an explosive expansion of the Internet of Things (IoT) [2], in which devices are connected to the world through the internet, has occurred. The data produced by people, machines, and IoT devices is forecasted, by the Cisco Global Cloud Index (GCI) [4], to exceed 500 Zettabytes (ZB) by 2020. To assist IoT devices in offloading their computation tasks, the cloud plays an important role in improving and expanding the capabilities of the IoT network by integrating the resource-constrained IoT with cloud computing [5,6,7]. Cloud-based IoT provides the resources of computational power, on-demand data storage, and offline analysis of massive amounts of data. However, cloud-based IoT currently faces various challenges in meeting the growing need for high performance. In particular, the dependence on cloud infrastructures can serve as a bottleneck for the latency and bandwidth requirements of applications [8]. When centralized cloud computing faces a large amount of user access, long-distance communication between users and cloud centers results in high service delays and wasted computer resources. The edge computing paradigm has emerged to achieve low latency, a bandwidth-efficient objective that not only enables processes but also the processing of large amounts of data at the edge of the network. The data can be analyzed near the edge of the network in the edge computing paradigm [9,10,11]. The edge of the network refers to units which are equipped with advanced computer platforms consisting of networking, storage, computing, and other core functions to produce/process data. There are several similar paradigms, such as fog computing [12] and mobile edge computing [13,14], which can offer effective solutions for mass data processing while improving the user experience.

The IoT has the ability to connect all of the things that surround us. To provide network services more reliably and accurately with IoT applications, heterogeneous devices are interconnected with each other, in order to gain and exchange sensed information between them through network infrastructures linked by distributed nodes [15]. The inherent characteristics of IoT, such as the large scale, the heterogeneity of the devices, and the large amount of data generated by the things, makes the development of a variety of applications and services a daunting task [16]. With limited network performance for data transfer, centralized cloud computing architectures have become increasingly inefficient in processing and analyzing the large amounts of data collected by IoT devices [17]. As edge computing makes computing resources available at the edge of the network (i.e., near to the devices generating the data), the bandwidth demands on the network performance are reduced [18]. Edge computing uses the resources of the connected devices to work together in achieving common goals in a distributed software architecture [19]. In the pursuit of the most suitable architecture for edge computing, modular characteristics relying on the microservice architecture have emerged [20]. The microservice approach of software architecture has become increasingly popular, due to its flexibility, granular approach, and loosely coupled services [21]. Microservices separate a complex application system into a small group of independent services. Each service is carried out as an independent process, performing only a specific task [22,23]. A microservice architecture allows developers to split an application into separate independent services, each with its own logic that can be managed and maintained by different development teams [9]. The microservice architectural style makes loose coupling, agility, and reuse possible, allows for horizontal scaling, and provides light services (instead of monoliths). The devices at the edge of an IoT network, as compared to a cloud server, have weaker processing power than a general-purpose computer and are limited by energy constraints. As a result, many attacks that may not work on desktop computers can pose a serious threat to edge devices [24]. In edge computing, the private data of the end-user must be outsourced by third parties, which may lead to data leakage and illegal data operations (such as replication and dissemination) and, so data confidentiality and integrity cannot be guaranteed [8,25]. Security experts have already warned that the potential risks are high with the number of unsecured devices that have been connecting to the internet since the concept of the IoT was initially proposed in the late 1990s. A researcher at Proofpoint, an enterprise security firm, discovered the first IoT botnet in December of 2013. More than 25% of botnets are composed of smart TVs, baby monitors, and other household appliances, as investigated by Proofpoint [26]. To protect the IoT devices (e.g., sensors and actuators) which are managed by a microservice architecture-based edge computing platform from illegal users, authentication-based methods have been investigated [26,27]. Authentication requires proof that the identity of the user is authorized to access the IoT devices.

In this paper, we aim to integrate edge computing platforms with an API gateway to provide edge computing clients with a secure authentication mechanism. We implement a microservice security agent based on the open-source Kong in the EdgeX Foundry framework in order to integrate an API gateway into the edge computing platform. The microservice security agent is responsible for authentication, authorization, and the redirect or route requests of the external clients to the endpoints of the internal microservices. The major contributions of this paper are as follows:

The API gateway acts as an intermediary between the external client and the microservices, providing a private network environment which allows for private data exchange among the microservices.

Authorization is implemented using JWT tokens, allowing access control enforcement in the API gateway to remove these concerns from the microservices, such that the microservices can remain lightweight to meet requirements of resource-constrained edge devices.

With a token-based authentication mechanism, the need for a client credential is replaced with a token that provides efficient client privacy preservation.

The rest of the paper is organized as follows. The related existing solutions are given in Section 2. In Section 3, the design of our proposed system configuration is presented. Section 4 describes the implementation environment. Section 5 describes with the performance of the proposed system. Section 6 concludes our work.

## 2. Related Work

In this section, we will survey the related works available in the literature. In [28], the possibility to report the power consumption data of customers to the control center in an authenticated and privacy-preserving way has been discussed. The authors used blind signatures and short, randomizable signatures to provide conditional anonymous authentication. To meet the aforementioned functions, they used powerful third parties to register entities and generate a certificate for each customer, control center, and fog nodes. However, the proposed solution consumed the computational power of resource-limited edge devices to generate secret keys from public and private keys.

The authors of [29] provided a secure and privacy-preserving mutual authentication solution for an Elliptic Curve Cryptography (ECC) fog-based publish-subscribe system. The proposed solution could ensure mutual authentication between subscribers and brokers, as well as between publishers and brokers. In addition, the proposed solution maintained user privacy, due to the difficulty of relating the requested topic for subscription and or publication to their anonymous identities and could withstand the curiosity of users and/or brokers. They showed that the proposed solution could ensure mutual authentication, confidentiality, anonymity, privacy-preservation, and message integrity. They used ECC to provide the same level of security with a lower key and message size than other public-key cryptography methods, such as RSA. To ensure confidentiality, anonymity, privacy-preservation, and message integrity, the publisher, broker, and subscriber used the private and public keys generated by the Trusted Authority (TA) to encrypt, decrypt, and calculate the secret keys. However, the proposed solution still consumed the computational power of the resource-limited edge devices.

The authors of [30] introduced three Lightweight Anonymous Authentication Protocols (LAAPs) to support three different Device to Device (D2D)-aided fog computing models. In this respect, they use lightweight cryptographic primitives, such as one-way functions and EXCLUSIVE-OR operations, which led to a limited computational cost for the resource-limited edge devices. They also introduced a novel privacy protection security architecture for the D2D-supported fog computing model, which allows end-user devices to be authenticated without the intervention of a central server. However, the proposed architecture and protocols are used to validate each edge device, network access device, and centralized cloud server. They did not consider user authentication, which is responsible for managing and maintaining the system.

Edge computing has been defined by the Edge Computing Consortium (ECC) [31] as an open platform deployed at the edge of the network near the data source and offering intelligent services for real-time processing, data optimization, security, and privacy within the mobile edge network infrastructure [32]. Edge computing can form a bridge between smart devices and cloud computing and storage services [33]. The edge of the network refers to units which are equipped with advanced computer platforms consisting of networking, storage, computing, and other core functions which produce data, as well as analyzing data to detect abnormal behaviors or failures in the connected smart devices. To cope with the above issues, a Lightweight Edge Gateway for the Internet of Things (LEGIoT) architecture [9] has been introduced, which is based on the modularity of microservices and the flexibility of simple virtualization technologies, in order to guarantee scalability and flexibility. In [34], the author presented an intelligent IoT gateway which can communicate with different networks, has a flexible protocol that converts different sensor data into a consistent format, and has a uniform external interface.

There exist several IoT platforms which provide a connection with IoT devices. Amazon has released the AWS (Amazon Web Services) IoT [35], which is a cloud platform. The goal of this framework is to allow IoT devices to easily connect to and securely interact with AWS cloud and other devices. There exist open-source client libraries and SDKs which make the AWS IoT framework available for embedded operating systems and microcontroller platforms. The programming language is C, Node.js, and the Arduino sketch. Ericsson [36] has released Calvin, an open-source IoT platform. The Calvin framework is designed for managing and building distributed applications to enable devices to interact with each other. It requires multiple programming languages, such as python, CalvinScript, and others. The data processing can be implemented with other languages, however. Kura [37] is an Eclipse IoT project whose goal is to provide an OSGI-based framework for IoT gateways. It provides a platform for managing communication between local networks of physical IoT devices, and is written in Java. The framework has requirements for the IoT devices, however: The operating system must be Linux-based. All the aforementioned IoT platforms have dependencies on certain operating systems or programming languages. The EdgeX Foundry project [38,39,40,41], however, has no dependencies on any operating system or programming languages. The EdgeX Foundry framework has been introduced by the Linux Foundation and Dell. It adopts a microservice architecture to deal with the edge computing paradigm. In EdgeX Foundry, all microservices are generally implemented as lightweight virtualization containers, which isolate microservices from each other and provide maintainability and scalability in the EdgeX Foundry framework.

Edge computing can provide storage and perform computational tasks at the edge of the network (instead of a data center in the cloud), which can create many security and privacy challenges. To deal with these problems, the authors of [42] proposed a method to provide a secure authentication mechanism for an IoT network which consists of several limited devices through a security manager offering authentication services for multiple IoT networks, which reduces the costs of managing a secure database in an IoT network. The authors of [43] recommended designing and implementing a token-based Message Queue Telemetry Transport (MQTT)—a popular messaging protocol in the IoT field—protocol authentication in an IoT network. The proposed design includes a publisher, a subscriber, a MQTT broker, and a token authentication server to cope with the security and scalability problems of using a MQTT protocol in the IoT network.

An API gateway [44] is an entry point for forwarding requests between many microservices, which merges multiple microservice APIs into a single client and and routes the requests from one access point to the correct microservices. The API gateway uses an existing identity management and authentication service which manages accounts, such as JWT or OAuth2.0 [45], to allow a user or client access to certain microservices. An API gateway [46] is a service that publishes multiple APIs, updates the published API set at runtime, and is integrated with service detection, load balancing, service monitoring, and security capabilities.

## 3. Microservice Security Design based on Token in Edge Computing

**Figure 1:**
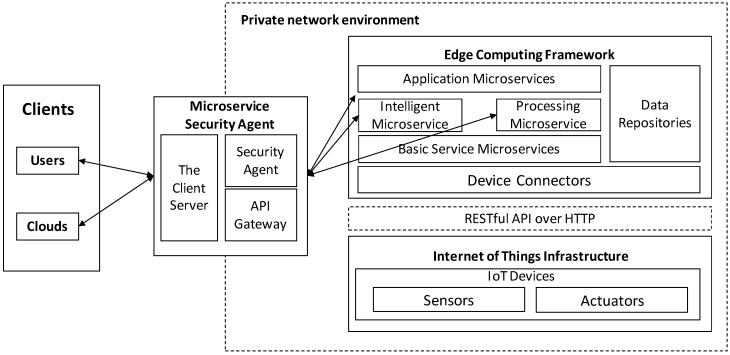
Edge Computing Environment Configuration.

We propose a microservice security agent based on an API gateway using tokens in an edge computing environment. As shown in Figure 1, the edge computing environment consists of a three-layer structure:

The client layer include clients, which are web browsers and mobile or centralized cloud computers that provide management functions for edge devices as consumers.

The edge computing framework layer consists of several fine-grained and self-contained microservices with individual functionalities, based on EdgeX Foundry. A microservice can be independently developed with individual technologies and deployed inside containers, which are portable, interchangeable, scalable, and lightweight execution environments. The edge computing framework decomposes centralized cloud computing services into minimal functional software modules, which are focused, self-contained services (e.g., basic service microservices, device connectors, data repositories, and other application microservices) to provide storage, intelligent processing, and internet capabilities; in contrast to cloud computing, in which the microservices provides services and communicate each other through a well-defined message interface, such as a REST API. From the point of view of the service consumer, the edge computing framework is a collection of APIs. To make it easier to access and manageme, we propose a microservice security agent as a microservice. The microservice security agent consists of three microservices: The client server is responsible for providing the Graphical User Interface (GUI) to the consumer. The API gateway runs in front of any microservices and is extended through plugins, which provide extra functionality, such as JSON Web Token (JWT) authentication. The security agent provides REST APIs to manage the API gateway microservice.

The Internet of Things infrastructure is constructed of various small single-board computers, sensing devices, and actuators. The IoT devices deployed in IoT infrastructures collect data from the physical world and operate the actuator to control electric devices. In general, the sensors and actuators are not capable of network functionality. Therefore, they are directly connected with IoT devices, such as a single-board computer, through a native interface. An IoT device is able to provide (wireless or wired) connection ability, in order to communicate with an edge computing framework, through IoT data protocols such as CoAP and HTTP.

**Figure 2:**
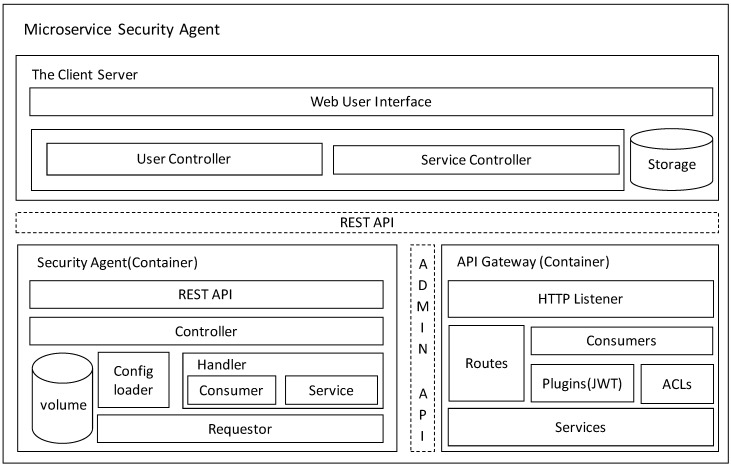
Configuration of the API gateway for microservice security.

The microservice security agent consists of three microservices, as illustrated in Figure 2. The client server is responsible for providing the Graphical User Interface to the client, with which users can be created and services provided by the edge computing framework can be consumed. It includes a web user interface (the GUI), where controllers receive requests and then generate responses, and storage is used to store the user information.

The API gateway, which is an open-source API gateway in Kong, runs in front of any REST API can be extended by plugins, which provide extra functionality such as JSON Web Token (JWT) authentication. The API gateway provides an HTTP listener to proxy incoming requests from clients and provide admin APIs for administration purposes. There are several components such as the service, which is the name the API gateway uses to refer to the microservice APIs it manages; the route, which makes requests against the service; the consumer, which is the user using the aforementioned service; and plugins, which extend Kong. For security concerns, we may add a JWT plugin to verify requests containing HS256- or RS256-signed JSON web tokens (as specified in RFC 7519).

The security agent provides essential functions to use the API gateway microservice. It provides REST APIs which the user can connect with, and consists of a controller, volume, config loader, handler, and requestor. The controller receives request from client and invokes a related function in response to the request. The volume includes a toml config file, which is the manifest of the microservices the API gateway manages, and a public key, which is used to generate the JWT certificate and verify JWTs with RS256 and ES256. The config loader parses specific configurations from the toml file, then creates related instances to provide information about microservices. The handler provides business logic to generate consumer-related requests and service-related requests, such as add service, route, and consumer, and enables JWT plugins to generate JWT certificates. The requestor is responsible for sending requests to the API gateway through the Admin API.

**Figure 3:**
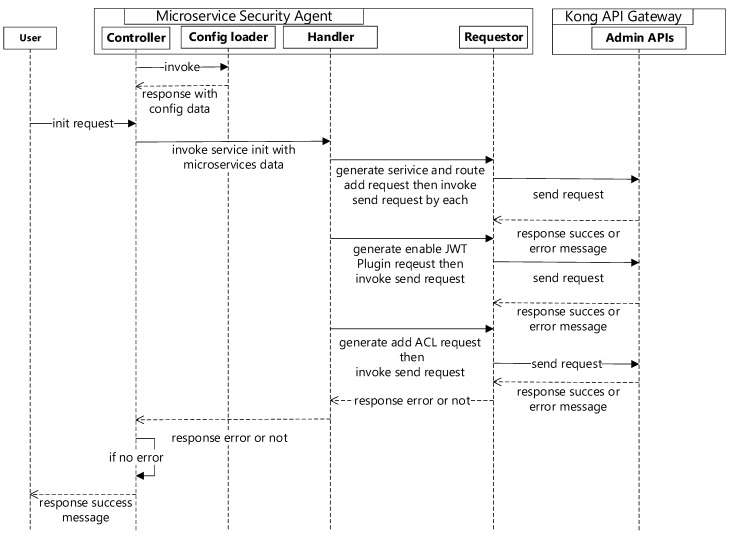
Sequence diagram for microservice security initialization.

To serve as microservices in the API gateway through Kong, we need to add microservice APIs as services in Kong. When an HTTP Get request is sent with the <Kong address:8000>/init API to the microservice security agent, first it invokes the config loader to parse the configuration file, then information is sent to a related structure in the Go language. Secondly, it invokes the handler to generate a request for adding the service and route, according to the information of the config file. Third, it sends a request through the requestor to the Kong API gateway admin API, after which the handler will enable generation of a JWT and an ACL list request, which are then sent to the Kong API gateway admin API to initialize the default services, routes, ACL, and authentication information. Figure 3 shows the sequence diagram for microservice security initialization.

**Figure 4:**
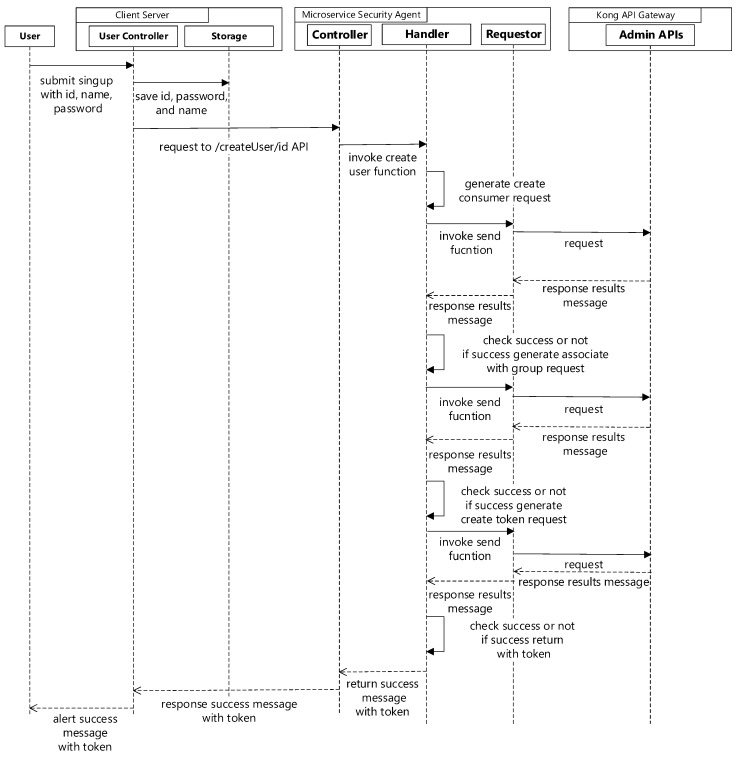
Sequence diagram for user creation.

When the ID, name, and password are submitted to the client server, it will save that information to storage (using MongoDB), then send a request to the microservice security agent to generate an associated consumer in the Kong API gateway, associate the consumer to a whitelist of a group, and enable JWT to generate a token. Figure 4 shows the sequence diagram for user creation.

**Figure 5:**
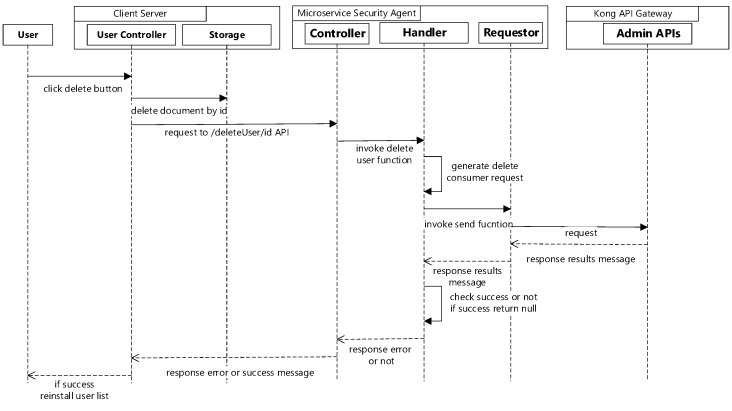
Sequence diagram for user removal.

When the delete button is selected on admin management page, the client server will delete the user information by ID, then send a request to the microservice security agent to delete the consumer in the Kong API gateway. The microservice security agent generates a delete request with the ID and the delete method sends it to the Kong API gateway. If the selected id of the consumer is successfully deleted in the Kong API gateway, the client server will reinstall the user list. Figure 5 shows the sequence diagram for user removal.

**Figure 6:**
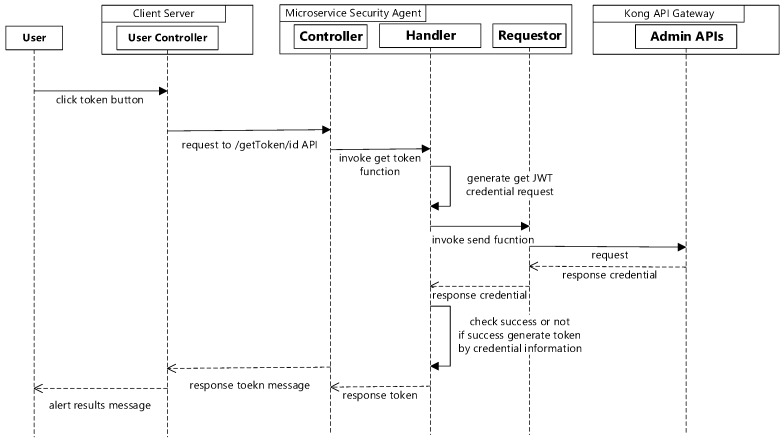
Sequence diagram for new token generation.

If the token button is selected on the admin page, the client server will send a request to the microservice security agent. The microservice security agent will generate a JWT get request with the ID of the selected user, then send it to Kong API gateway. It then parses the response of the Kong API gateway to generate a token with the credentials. The token will be alerted by the client server. Figure 6 shows the sequence diagram for new token generation.

**Figure 7:**
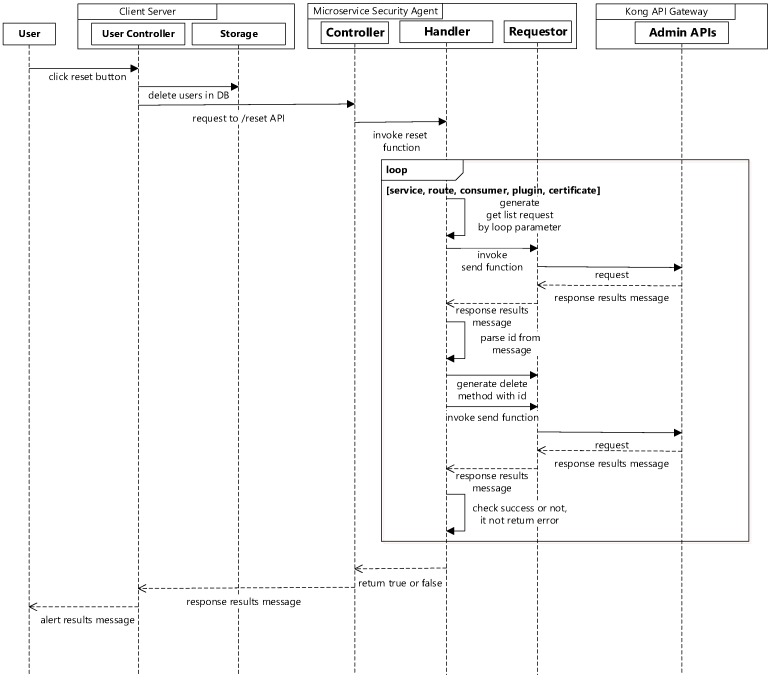
Reset sequence diagram for removing all the configurations.

If it is necessary to remove all the information registered in the Kong API gateway, there is a reset button in the client server. When it is selected, the client server will send a request to the microservice security agent. The microservice security agent will retrieve the IDs of the services, routes, consumers, plugins, and certificates and generate a delete request for all of them. The requests will be sent to the Kong API gateway to remove the related features in it. Figure 7 shows the sequence diagram for removal of all configurations in the Kong API gateway.

## 4. Implementation

To develop the proposed solution, different types of software and hardware components are needed. The hardware we used was a desktop computer with the Ubuntu 18.04 operating system, an AMD 64 quad core, 4 GB memory, and a 100 GB hard disk. For software, we used Docker and Docker Compose to ease the operation of the applications, including the open-source microservice Kong API gateway. The details of the hardware and software specifications are presented in Table 1.

To provide an easy-to-use approach with Kong, we implemented REST APIs for communication with Kong (see Table 2). The Kong microservice API gateway provides admin REST APIs for administration purposes. To proxy the microservice APIs securely, we needed to set up several things, such as services, routes, consumers, plugins, and an ACL (access control list). The services are the names that Kong uses to refer to the upstream APIs and microservice APIs, in order to provide such services to the clients it manages. The routes are mapping rules that specify how requests are sent to their service, to map requests from client to pre-added services. The consumers are associated with individuals using the services, which can be used for access management. The plugins allow for easily adding new features to services; for security concerns, we added a JWT plugin to verify requests containing HS256- or RS256-signed JSON Web Tokens (as specified in RFC 7519). Each consumer has individual JWT credentials (i.e., public and secret keys), which must be used to generate their JWTs. It is annoying and tedious to set this up for all microservices APIs repeatedly. Therefore, we implemented a microservice security agent for the admin REST APIs of Kong as a Docker image to reduce client effort and simplify deployment and operations costs. Registration of a microservice API to Kong is done through an HTTP get request to the <Kong address:8000>/init. Each piece of service-related information, such as the name of the microservice, host, port, and protocol, is stored in toml files. According to the configuration file, the agent will add services along with its routes, plugins, and ACL. Consumer registration is done through an HTTP Post request to the <Kong address:8000>/createUser with the “user” and “group” keys of the JSON body. User is the consumer name group, which is the name in the ACL. It returns the token generated by the JWT credentials of the consumer with public and secret keys. To obtain the token with an existing consumer, it also provides an API <Kong address:8000>/getToken with a get method. There are also other APIs for deleting registered consumer and services. Finally, it provides an API <Kong address:8000>/ for monitoring the status of Kong.

**Figure 8:**
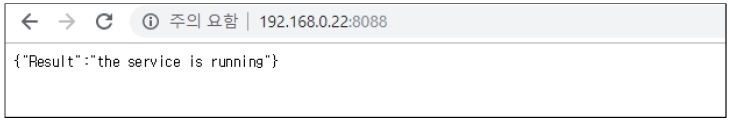
Result of monitoring the Kong API gateway using the root API.

When sending a request to the microservice security agent with the API “/”, it will respond with the status of the Kong API gateway. Figure 8 shows the response when the status of the Kong API gateway service is “running”. As the Kong agent and the proposed system depend on the Kong API gateway, the status of the Kong API gateway is important to monitor.

**Figure 9:**
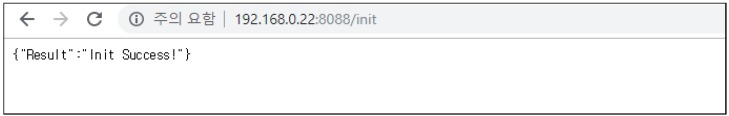
Result of initializing the Kong API gateway using the init API.

The result of running the microservice security agent API “/init” is shown in Figure 9. If the microservice API-related services, routes, and plugins are successfully initialized, a success message is returned. Before the microservices are started in the proposed system, the Kong API gateway must be initialized; when it is successfully initialized, the microservices can serve as services securely.

**Figure 10:**
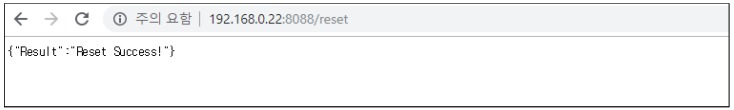
Result of resetting the Kong API gateway using the reset API.

The API “/reset” is used for reconfiguring the microservice security agent. It cancels all settings of the microservice security agent in one call. Figure 10 shows the response of the API to the reset API.

**Figure 11:**
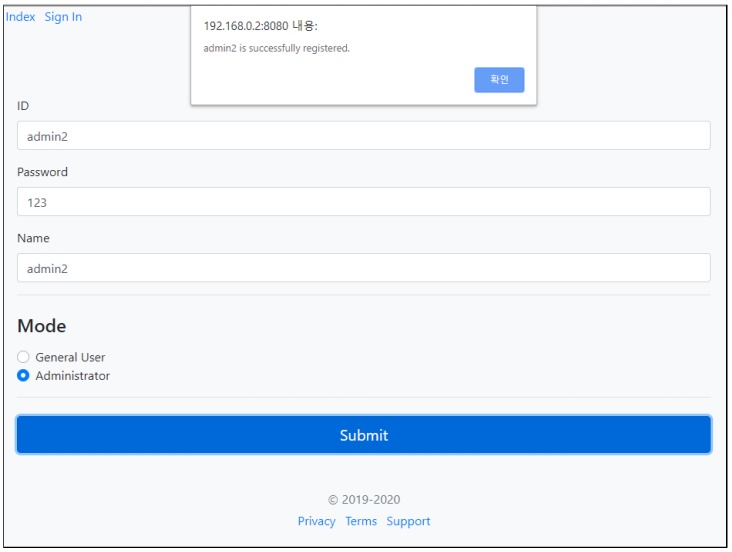
Result of creating a user using the createUser API.

To make it easier to visit the API “/createUser”, we implemented a Graphical User Interface with ID, password, and name fields. A user with administrator privilege can be created with ID and password, where the name is displayed. When the credentials are submitted with complete information, a request will be sent to the API to create a consumer through the microservice security agent. The received response message will then be parsed to obtain a token to store the information in MongoDB with the related user information. If there is no error, the GUI will return with a success alert. Figure 11 shows the GUI and the result of successfully creating a user.

**Figure 12:**
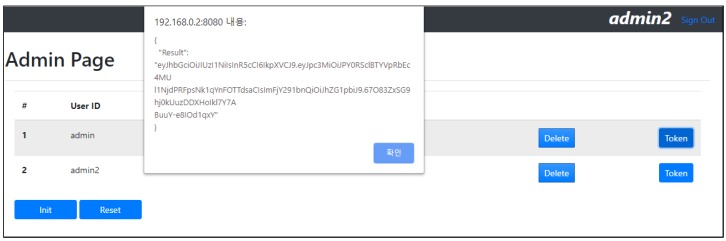
Result of obtaining a token using the getToken API.

There is also an admin page to manage all users generated with the microservice security agent. For demonstration purposes, we implemented a button to trigger the “/getToken/user” API to send a request to the microservice security agent. Figure 12 shows the result of the API “/getToken/user”, which shows a returned token with the key “Result”.

## 5. Performance Analysis

To assess the practical applicability of the designed system, we analyzed the round trip time (RTT) in practice. We compared the measured RTT time with the reaction time [47,48], the measure of quickness with which an organism responds to some sort of stimulus. If the RTT time is less than the reaction time, the proposed system is applicable within the real world. The statistical average reaction time, obtained from 81 million records, is 284 milliseconds (i.e., 0.284 s).

**Figure 13:**
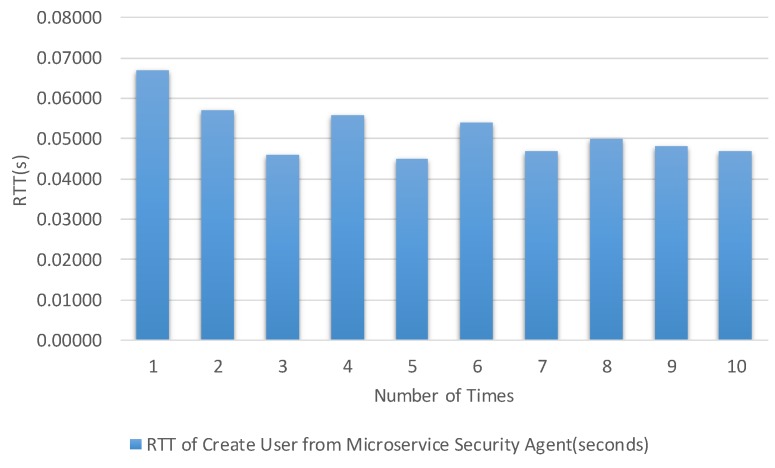
Round trip time for creating a user based on the create user API.

**Figure 14:**
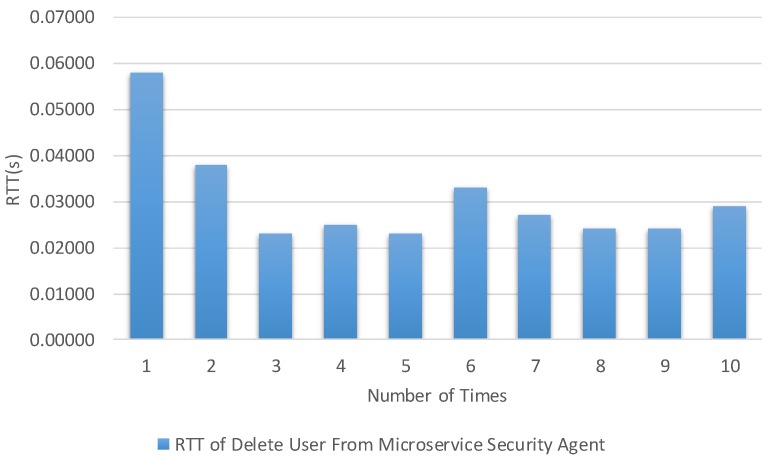
Round trip time for deleting a user based on the delete API for user removal.

**Figure 15:**
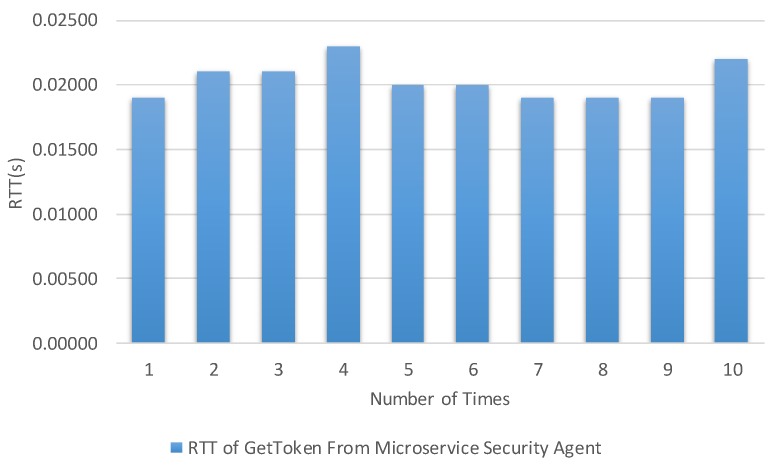
Round trip time for obtaining a token based on the getToken API.

**Figure 16:**
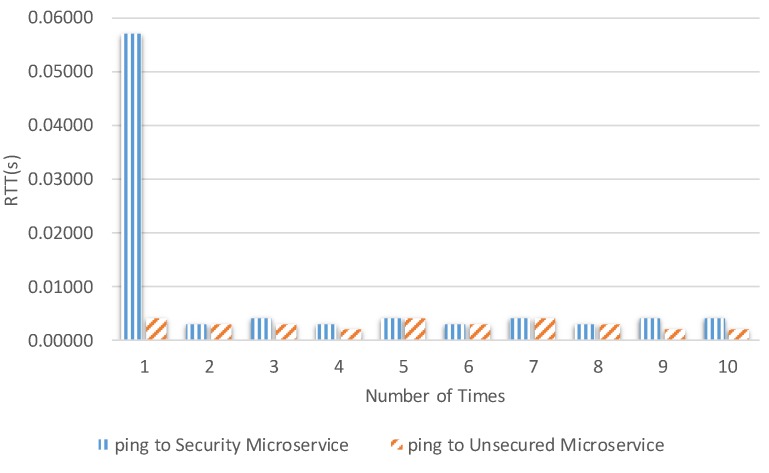
Round trip time for using the microservices API, with and without a token.

The times were calculated from the client to the microservice security agent and internal microservice. Figure 13, Figure 14, Figure 15 and Figure 16 show the results of our experiment for creating/deleting a user, generating a token, and sending a request to the microservice with and without a token, respectively. The specific results are summarized in Table 3 in the second column.

The authors of [43] measured the performance of the proposed system based on the time it took for the publisher/subscriber to obtain the token from the authentication server; the average time was below 1 s. As can be seen in Figure 15, the average time to obtain a token from the microservice agent was less than 0.1 s. This indicates that our solution is better than the existing solution.

## 6. Conclusions

Edge computing integrates modular characteristics reliant on microservices to provide an extensible and lightweight computing framework at the edge of a network to achieve low latency, a bandwidth-efficient objective which not only produces data, but can also analyze large amounts of data at the edge of the network. However, compromised smart objects not only lead to data leakage, but can also result in physical threats to users, as IoT aims to connect all of the things which surround us. This means that security is still a fundamental problem in edge computing. In this paper, we have presented a method to secure microservices based on tokens using a REST API gateway in the edge computing environment as a microservice to provide a lightweight and secure computing framework at the edge of the network. We have shown how, by means of an API gateway service, it is possible to provide a secure IoT at the edge of a network. We have evaluated our approach by comparing the measured RTT time with the reaction time (the measure of quickness of an organism to respond to some sort of stimulus). As a result, we conclude that the proposed system is suitable for real-world applications.

References

## Figures and Tables

**Table 1:** Hardware and software specifications for microservice security.

Category	Item	Specification	Description
Hardware	Desktop	OS	Ubuntu 18.04 Desktop
		CPU	AMD 64 quad core
		Memory	4 GB
		Hard Disk	100 GB
Software	Library	Go language	An open source programming language.
	Application	Docker, Docker Compose	A platform for developers and system admins to develop, ship, and run applications.

**Table 2:** Specifications of the microservice security agent REST APIs.

API	Method	Path Variable	Body	Remarks
/	Get	No	No	Get the status of the Kong API gateway.
/init	Get	No	No	Initialize the Kong API gateway with services, routes, ACL, and plugins.
/reset	Get	No	No	Delete all init items, including those added (consumers).
/getToken/{user}	Get	{user}	No	Get the token by name.
/createUser	Post	No	JSON {“user”:”<user>”, “group”:”<group>”}	Create consumer and associated group of ACL and return token.
/deleteUser/{user}	Delete	{user}	No	Delete consumer from Kong.

**Table 3:** Round trip time test results.

Test Item	Maximal	Minimal	Average Time
Round trip time for creating a user based on the create user API.	0.067 s	0.045 s	0.0517 s
Round trip time for deleting a user based on the delete API for user removal.	0.058 s	0.023 s	0.0304 s
Round trip time for obtaining a token based on the getToken API.	0.023 s	0.019 s	0.0203 s
Round trip time for using the microservices API with a token.	1.057 s	0.003 s	0.0089 s
Round trip time for using the microservices API without a token.	0.004 s	0.002 s	0.003 s
